# The distributional effects of tobacco tax increases across regions in Mexico: an extended cost-effectiveness analysis

**DOI:** 10.1186/s12939-021-01603-2

**Published:** 2022-01-20

**Authors:** Belen Saenz-de-Miera, Daphne C. Wu, Beverly M. Essue, Norman Maldonado, Prabhat Jha, Luz Myriam Reynales-Shigematsu

**Affiliations:** 1 Department of Economics, Universidad Autonoma de Baja California Sur, Carretera al Sur Km 5.5, 23080 La Paz, Baja California Sur Mexico; 2 Centre for Global Health Research, Unity Health Toronto, University of Toronto, Toronto, Canada; 3 PROESA, Universidad Icesi, Cali, Colombia; 4 Departamento de Investigacion sobre Tabaco, Instituto Nacional de Salud Publica, Cuernavaca, Mexico

**Keywords:** Tobacco taxation, Distributional effects, Subnational, Mexico

## Abstract

**Background:**

Several studies have shown the beneficial effects of tobacco fiscal policy, but distributional effects have been less examined, especially at the subnational level. The objective of this study is to analyse the distributional effects of a one-peso tobacco tax increase (roughly equivalent to tripling the current excise tax) on health, poverty, and financial outcomes at the subnational level in Mexico.

**Methods:**

We employ an extended cost-effectiveness analysis that estimates life-years gained, smoking attributable deaths averted, treatment costs averted, number of persons avoiding poverty and catastrophic health expenditures, and additional tax revenues by income group across five regions.

**Results:**

With the one-peso tax increase (or 44% price increase), about 1.5 million smokers would quit smoking across the five regions, resulting in nearly 630 thousand premature deaths averted and 12.6 million life years gained. The bottom income quintile would gain three times more life years gains than the top quintile (ratio 3:1), and the largest gain for the most deprived would occur in the South (ratio 19:1), the region with the highest poverty incidence. Costs averted and additional tax revenues would reach 44.6 and 16.2 billion pesos, respectively. Moreover, 251 thousand individuals would avoid falling into poverty, including 53.2 in the lowest income quintile, and 563.9 thousand would avoid catastrophic health expenditures. Overall, the bottom income group would obtain 26% of the life years gained and 24% of the cost averted, while only paying 3% of the additional tax revenue.

**Conclusions:**

The most significant gains from a substantial cigarette price increase would be for the poorest 20%, especially in the South, the most impoverished region of Mexico. Therefore, tobacco taxes are an opportunity for governments to advance in equity and towards the achievement of sustainable development goals on non-communicable diseases.

**Supplementary Information:**

The online version contains supplementary material available at 10.1186/s12939-021-01603-2.

## Background

Despite significant progress, 15 years after establishing the WHO Framework Convention on Tobacco Control (WHO-FCTC) [1], tobacco use remains the main preventable risk factor for death and disability globally [2]. The epidemic has spread to low- and middle-income countries, which host the largest number of smokers and face all the negative consequences on morbidity and mortality, as well as the burden for households and the health system of health care expenditure for tobacco related illnesses [2, 3]. In Mexico, 17% of the adult population currently smokes (i.e., 14 million smokers) [4], and 58,200 tobacco-attributable deaths occur annually, mainly due to cardiovascular diseases, chronic respiratory diseases, and lung cancer [5]. These diseases also impose a significant burden on the Mexican health system that amounts to about 4800 million 2015 US dollars per year (or 75,600 million pesos) [6]. Importantly, a considerable part of this double health and economic burden could be avoided if cost-effective measures were implemented to reduce tobacco consumption.

Mexico was the first country in the Americas to ratify the WHO-FCTC in 2004, and has implemented several tobacco control measures at the national and subnational level, such as graphic warnings, restrictions on tobacco advertising, complete ban on tobacco-product sponsorships and promotional items, and smoke-free indoor environments with special smoking areas —except for 15 states that fully protect non-smokers [7]. In particular, the structure of the tobacco excise tax (called Special Tax on Production and Services or IEPS by its acronym in Spanish) has been significantly strengthened with the homologation of rates for all tobacco products in 2007 (except for those entirely handmade) and the incorporation of a specific component of 4 cents per cigarette in 2010 which was substantially increased to 35 cents in 2011 [8]. The results of this large increase demonstrated the beneficial impact of taxation on tobacco consumption and revenues [9]. More recently, the specific component was raised to 49.44 cents to account for accumulated inflation between 2011 and 2019, and its annual indexation to the consumer price index was approved. Although the overall prevalence of smoking decreased 11.2% or 2.4 percentage points between 2002 and 2016 (from 21.4 to 19.0%) —mainly due to reductions among daily smokers (from 13.5 to 7.0%)— [10], a substantial tax increase is needed to further reduce smoking prevalence to less than 12.5% by 2025 (the 30% reduction target relative to 2010) [11], demonstrating that taxes are a central public health measure to cut down the epidemic of noncommunicable diseases and advance towards the achievement of goal three of the 2030 Agenda for Sustainable Development [12, 13].

While there is ample evidence on the effectiveness of tobacco taxation to promote quitting, reduce consumption and discourage initiation, [14, 15] concerns about its distributional effects frequently arise when proposals to strengthen fiscal policies are discussed. A recent study of the Global Tobacco Economics Consortium (GTEC) in 13 middle-income countries showed that a 50% tobacco price increase would strongly favour those in the bottom income group [16]. Specifically, they would obtain 31% of the life years gained and 29% of the averted costs, while only paying 10% of the additional taxes. Using a similar approach for eight low- and middle-income countries, Fuchs et al. also showed that tax increases are largely pro-poor [17]. However, the evidence is still limited and, especially, within-country variations have been ignored. Often, countries present significant geographical differences in the tobacco epidemic and health system coverage that should be considered to better understand inequalities in health across regions and the potential contribution of tobacco taxes to address those inequalities. From a policy perspective, this information could offer powerful arguments to gain support from sub-national stakeholders to move forward in tobacco taxation. Hence, the aim of this study is to extend previous analyses to estimate the distributional effects of tobacco fiscal policy at the subnational level in Mexico.

Mexico provides an ideal setting for regional analyses of the distributional effects of tobacco taxation for two main reasons. First, there are important variations in the tobacco epidemic at the subnational level that may be masked in national analyses [18]. In general, the highest smoking prevalence is observed in the northeast. However, the Centre has the highest concentration of smokers, while the South has the highest concentration of low-income smokers. Second, due to these variations in the epidemic there are significant differences in the magnitude of the burden of disease (morbidity and mortality) and treatment costs attributable to tobacco across regions [19, 20]. For example, the Northwest has relatively more lung cancer deaths attributable to tobacco. The overall effect of higher taxation across income groups importantly depends on averted medical costs, which tend to favour the poorest. For this study, we divided the country into five regions to analyse the health and financial effects across income quintiles of a substantial one-peso tax increase, which is roughly equivalent to tripling the current specific component of the tobacco excise tax.

The rest of the paper is organised as follows. The second section provides a detailed description of the model and data sources; the third section presents the results, including some sensitivity analyses; the fourth section discusses the findings; and conclusions are presented at the end.

## Methods

### The model

We employ the extended cost-effectiveness analysis (ECEA) model recently used by the GTEC to evaluate the cumulative health and financial effects of a 50% price increase across income groups in 13 middle-income countries, including Mexico [16]. Specifically, this compartmental model estimates, by income quintile, life-years gained, smoking attributable deaths averted, treatment costs averted, number of persons avoiding poverty and catastrophic health expenditures, and additional tax revenues that would result from tobacco excise tax increases. While other simulation models have provided evidence on the potential effects of a substantial tobacco tax increase in Mexico, none of them have calculated distributional impacts [6, 21].

The model first determines the number of smokers in the current smoking cohort who would quit due to a tax increase, which depends on the price elasticity of demand by income group. Then, based on the benefits of quitting, total life years gained and tobacco attributable deaths averted are estimated. Treatment costs averted are derived from the latter. Specifically, total deaths averted are apportioned across the four main causes of smoking attributable mortality (chronic obstructive pulmonary disease (COPD), heart disease, stroke and lung cancer), for which the annual treatment cost is known. The persons avoiding poverty are those whose income does not fall below the poverty line because the tax prevents out-of-pocket expenses that would otherwise be necessary to cover treatment costs of smoking attributable diseases. Similarly, the persons avoiding catastrophic health expenditures are those whose out-of-pocket health expenditure does not exceed 10% of their yearly income as a result of averted treatment costs. Finally, the additional tax revenue collected is calculated with the price per pack, the tobacco excise tax as a share of price, and average cigarette consumption. The time horizon for the estimates is therefore the lifetime of the current smoking cohort (from baseline, 2020, until the last person in the cohort dies), except for the additional tax revenue that is annual. More detail on the theoretical foundation and statistical procedures can be found elsewhere [16].

**Fig. 1:**
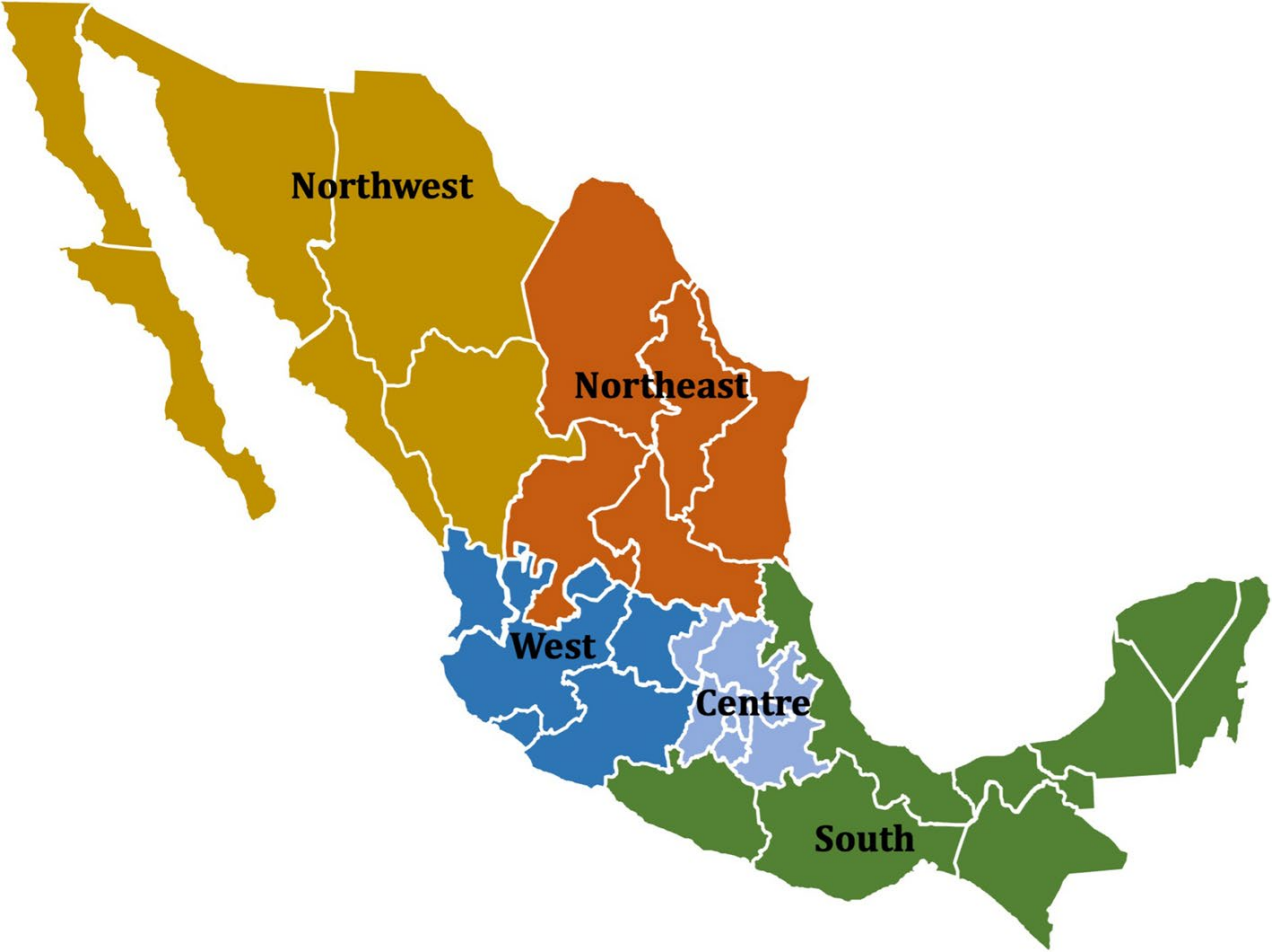
Regions considered to analyse the impact of a one-peso tobacco tax increase on health and financing outcomes in Mexico

To study the effects of a tax increase at the subnational level, the country was divided into five regions (Fig. 1): the Northwest region that comprises the states of Baja California, Baja California Sur, Sonora, Sinaloa, Chihuahua, and Durango; the Northeast region that comprises Coahuila, Nuevo Leon, Tamaulipas, San Luis Potosi, and Zacatecas; the West region that comprises Nayarit, Jalisco, Michoacan, Colima, Aguascalientes, and Guanajuato; the Centre (or Megalopolis) that comprises Mexico City, Estado de Mexico, Hidalgo, Morelos, Puebla, Queretaro, and Tlaxcala; and the South region that comprises Guerrero, Oaxaca, Veracruz, Tabasco, Chiapas, Campeche, Quintana Roo, and Yucatan. The definition of these regions not only took into account geographic proximity among states, but also epidemiological and economic conditions. The southern region, which has relatively few smokers, was not further divided as the sample to estimate key epidemiological parameters would have lacked statistical power (i.e., the sample size to estimate these inputs was prioritized over the other criteria).

We consider both male and female smokers, as the latter constitute 28% (4.2 million) of all adult smokers 20 years and older. Similarly, we consider not only daily but also occasional smokers, as more than half of all adult smokers smoke occasionally (57% of all adult smokers or 8.4 million).

The modelled scenario considers a one-time, one-peso (PPP$0.108) increase in the specific component of the tobacco excise tax, which is currently 0.4944 (PPP $0.053) pesos per cigarette. This increase from 0.4944 to 1.4944 pesos would raise retail prices by 43.9% on average (from 56.4 to 81.2 pesos per cigarette pack), considering a full pass-through to the consumer [22]. The tobacco excise tax incidence would increase from 55.4% of the retail price to 64.1% on average (Supplementary Table A1). This level of increase has already been considered in recent initiatives presented in Congress and is in line with international recommendations to achieve the 2030 Agenda goal of reducing premature deaths from noncommunicable diseases by 30% [23, 24]. Sensitivity analyses, however, consider a 1.15 pesos tax increase (from 0.4944 to 1.6444 pesos) that corresponds to a 50% price increase. This alternative scenario has been considered in other simulation models and previous versions of the GTEC ECEA [ 6, 16, 21].

To calibrate the model, the tax revenue estimate for the base year was adjusted to match actual revenue (Supplementary Table A2). Since the latter has been consistently reported for decades by the Ministry of Finance (SHCP) and the excise tax per cigarette pack comes from direct observation of prices and the tax structure established by law (see section 2.2 below), discrepancies between the estimated and the actual revenue at baseline were assumed to come from underreporting in the quantity consumed. In other words, we maintained the distribution of average consumption across quintiles and regions derived from national surveys (see section 2.2 below) but adjusted the quantity upwards. This procedure to estimate total consumption based on tax revenue and cigarette taxes per pack yielded similar results to those from other sources for recent years [25].

All the analyses were conducted using Stata 16 [26].

### Parameters and data sources

Supplementary Table A3 shows the model parameters at the national level and for each region. If subnational information was not available (e.g., treatment costs), national parameters were used for all regions. Definitions and data sources are specified below.

#### Number of smokers

The number of smokers by six age groups (10–14, 15–24, 25–44, 45–64, 65–84, and 85 years and more) and income quintiles (where the first quintile corresponds to the poorest 20% of the population) was calculated directly from the most recent national health survey, the National Health and Nutrition Survey 2018–19 (ENSANUT), to ensure consistency between the sum of the regional figures and national estimates for this parameter. ENSANUT is the main source of information to monitor the health and nutritional status of the Mexican population, as well as the performance of health services. The last round of 2018–19 is representative at the national and state levels, which makes it ideal for subnational estimates. ENSANUT employs probabilistic, multi-stage sampling procedures to select households for face-to-face interviews; up to one adult 20 years or older and one adolescent 10 to 19 years are interviewed per household. The sample consists of 60,995 respondents aged 10 years and older. Except for the age groups in the extremes, coefficients of variation were generally below 20, suggesting the power of the sample was acceptable to conduct the estimates at the regional level. It was assumed that there were no smokers among those under 10 years. The tobacco section of ENSANUT 2018–19 includes ten questions that have been harmonised with those of the Global Adult Tobacco Survey (GATS), specifically designed to measure the tobacco epidemic based on internationally standardised definitions [27]. Smokers are respondents who reported currently smoking tobacco, either daily or occasionally. Since ENSANUT does not collect household income, a proxy was defined using principal component analysis and information on household durable items and housing characteristics. The thresholds to define quintiles were set at the national level. ENSANUT 2018–19 data and documentation are public through the microsite created by the National Institute of Public Health (INSP), responsible for the survey together with the National Institute of Statistics and Geography (INEGI) [28].

#### Cigarette consumption

Cigarette consumption by income quintile was also estimated from ENSANUT 2018–19. Daily smokers report average daily cigarette consumption, while occasional smokers report average weekly cigarette consumption. The latter was simply divided by seven to obtain average daily cigarette consumption for all smokers.

#### Disease share of total deaths

Smoking-attributable mortality comes from the Global Burden of Disease (GBD) database that includes state level information for Mexico [29]. The GBD study is the most comprehensive source of information on causes of death, diseases and injuries, and risk factors worldwide. All data inputs, analyses, methods, and results are public. Total deaths are the sum of all smoking-attributable deaths from the four diseases considered, for population aged 30 years and over. This parameter is simply the share that corresponds to each disease. Regional estimates result from the aggregation of state level figures.

#### Risk reduction

Age-specific benefits from quitting come from studies in high- and middle-income countries used in earlier estimates of the model [16]. According to these studies, smoking is responsible for the deaths of at least half the current smokers who initiate early and do not quit [30], whereas avoided excess mortality ranges from 97% for those who quit at the 15–24 years age group to 25% for those who quit at the 65–84 age group [31, 32].

#### Life years gained

Age-specific total life years gained from quitting also come from studies in high-income countries employed in earlier estimates of the model [16]. In particular, 10, 9, 6 and 3 years gained for cessation before 30, 30–44, 45–64, and more than 65, respectively, are considered.

#### Health care utilisation

Health care utilisation was defined among ENSANUT 2018–19 respondents who reported having a health problem within the 2 weeks prior to interview. Specifically, we calculated the share of these respondents who sought professional health care by income quintile. These estimates were then expressed relative to the middle-income group (i.e., Q3 = 1) to capture disparities in utilisation conditional on being ill across quintiles.

#### Probability of seeking health care

The probability of seeking health care conditional on having any of the four diseases considered came from previous estimates of the model [16].

#### Health insurance coverage

To measure insurance coverage, ENSANUT 2018–19 respondents in each income quintile were classified in three groups based on whether they reported having access to health services provided by: 1) social security institutions (IMSS, ISSSTE, Pemex, Defensa, Marina), 2) Seguro Popular (SP, recently replaced by the Institute of Health for Welfare or INSABI), or 3) none (uninsured). Social security institutions provide health care access to formal sector workers and their families (nearly half of the population), while SP was created in the first half of the 2000s to provide health care access to informal sector workers and their families. Very few individuals reported having private health insurance (less than 1%); these cases were included in the first group, since private insurance coverage is more likely to resemble that of social security. Insurance coverage was disaggregated by area of residence, as rates importantly vary across urban and rural areas.

#### Insurance financial protection

Since social security covers, in principle, the total cost of the medical treatment for the four diseases considered, the beneficiaries of these institutions have full financial protection. On the other hand, SP (now INSABI) only covers the complete treatment of COPD. For the other three diseases, we assumed that beneficiaries pay fees that depend on socioeconomic status, as is done in INCAN [33]. For example, patients in the bottom socioeconomic group contribute 68% of the cost of cancer treatment so financial protection in that case is 32%. Finally, we assumed that those who report no insurance coverage pay the full treatment of the four diseases, i.e., financial protection is nil in these cases. However, we considered an additional scenario in which the uninsured have the same financial protection of SP beneficiaries. The rationale for this alternative scenario is that since the creation of INSABI in 2020, no affiliation is required. In other words, all uninsured are automatically entitled to the benefits previously offered by SP. In the future, INSABI is expected to cover the treatment of all diseases, including high-cost interventions, but it currently operates based on the SP package.

#### Treatment costs

Treatment costs for the four diseases considered are from previous studies on health care costs attributable to smoking in Mexico that have been employed in other simulation models [6, 16]. These figures were brought to current pesos using the Consumer Price Index (INPC). Unfortunately, there are no estimates at the subnational level, so national averages were employed for all regions. These estimates considered the main units of public health care providers that cover most of the population (social security institutions and the Ministry of Health, with more than 80% coverage; Table 1) and treat the main diseases attributable to tobacco in Mexico [34, 35]. In the case of IMSS, the principal health care provider, the costs were estimated with a national perspective from a sample of clinical records from 12 delegations, which allowed estimating the frequency of use by disease, severity, level of attention, and region. National averages were weighted according to these frequencies to capture such variability.

**Table 1:** Key epidemiological and economic indicators by region

Indicators	Regional					National
	Northwest	Northeast	West	Centre	South	
Population (in thousands, 2019)^a^	16,058	16,738	22,638	41,320	29,206	125,960
Smoking prevalence by income group (%, individuals 10 + years, 2019)^b^						
First (bottom 20%)	20.7%	16.3%	16.3%	13.7%	8.9%	12.1%
Second	18.7%	18.1%	19.2%	15.3%	10.5%	15.2%
Third	17.0%	18.0%	17.3%	17.3%	9.7%	16.1%
Fourth	15.6%	18.5%	16.1%	18.7%	12.6%	16.8%
Fifth (top 20%)	14.8%	18.1%	15.2%	16.5%	11.9%	15.9%
Total	16.6%	18.0%	16.6%	16.5%	10.1%	15.3%
Number of smokers (in thousands, individuals 10 + years, 2019)^b^						
First (bottom 20%)	266	214	277	608	879	2245
Second	409	366	556	1059	563	2952
Third	506	517	707	1226	346	3301
Fourth	518	610	726	1307	367	3529
Fifth (top 20%)	586	824	791	1634	257	4091
Total	2284	2531	3057	5833	2413	16,100
Average cigarette consumption per day (2019)^b^	4.3	3.4	4.6	2.7	1.7	3.2
% of urban population (2019)^b^	83.1%	84.2%	77.8%	84.7%	61.4%	77.9%
Population in poverty (%, in thousands, 2018)^c^	27.6% (4496)	28.7% (4736)	36.5% (8125)	42.5% (17,542)	60.9% (17,526)	41.9%(52,426)
Population in extreme poverty (%, in thousands, 2018)^c^	2.3% (375)	2.8% (454)	4.0% (892)	4.8% (1982)	19.5% (5607)	7.4% (9310)
GDP per capita (MX$, 2018)^d^	205,545	238,552	163,539	186,512	128,332	178,221
Public health expenditure per capita (MX$, 2018)^e^	5460	4987	4514	5845	4430	5115
Health insurance coverage (%, 2019)^b^						
Social security	59.8%	59.8%	47.2%	43.3%	30.5%	45.5%
Seguro Popular	24.2%	26.6%	34.8%	34.0%	52.0%	35.9%

Northwest = Baja California, Baja California Sur, Chihuahua, Durango, Sonora and Sinaloa. Northeast = Coahuila, Nuevo León, San Luis Potosí, Tamaulipas and Zacatecas. West = Aguascalientes, Colima, Guanajuato, Jalisco, Michoacán and Nayarit. Centre = Ciudad de México, Estado de México, Hidalgo, Morelos, Puebla, Querétaro and Tlaxcala. South = Campeche, Chiapas, Guerrero, Oaxaca, Quintana Roo, Tabasco, Veracruz and Yucatán

*GDP* Gross Domestic Product, *MX$* Mexican pesos

^a^CONAPO

^b^ENSANUT 2018–19 (INEGI/INSP)

^c^CONEVAL

^d^INEGI

^e^Ministry of Health

#### Poverty line

The income poverty line is from the National Council for the Evaluation of Social Development Policy (CONEVAL), the public institution that establishes the guidelines and criteria for poverty measurement in Mexico. The poverty line employed is equivalent to the value of the food basket per person, per month, in urban areas [36]. The rural poverty line was not used because this distinction would require disaggregated information on treatments costs and insurance financial protection across area of residence and, unfortunately, such information is not available. The main implication of using the urban poverty line exclusively is that the poverty headcount ratio at baseline is slightly overestimated (17% in this study compared to 14% according to CONEVAL), so that the estimates of poverty avoided due to the tax increase can be considered conservative. International poverty lines from the World Bank were also considered [37].

#### Per capita annual household income

Income data are from the National Survey of Household Income and Expenditure (ENIGH). The ENIGH was conducted twice in the 1980s and then every 2 years since 1992 —except for a special round implemented in 2005. This survey is employed to estimate the weights for the Consumer Price Index and official poverty figures, among other important indicators. While the last two rounds of 2016 and 2018 are not comparable with previous rounds due to relevant methodological modifications, they are not only nationally representative but also allow estimates at the state level. The sample of the ENIGH 2018 employed to determine household per capita income is composed of 74,647 household level observations and 269,206 individual level observations. Total current per capita income was defined following CONEVAL, so that poverty estimates at baseline closely correspond to the official estimates (see poverty line definition above). Income quintiles were assumed to be roughly equivalent to those defined with ENSANUT. ENIGH data and documentation are publicly available on the INEGI’s website [38]; do files and methodological documents from CONEVAL are also publicly available [36].

#### Cigarette price per pack

The cigarette price per pack is a weighted average of cigarette prices from INEGI. Specifically, INEGI collects monthly data of several brands in 46 cities as part of the information required to estimate the CPI to measure inflation in Mexico. Only prices of 20-cigarette packs —the most common— were considered. This information is not intended to be representative at the subnational level, so only a national average was calculated. However, since the tobacco excise tax is uniform, prices of legal brands are similar throughout the country. Market shares by brand were employed as weights [39].

#### Tobacco excise tax as a share of price

The average excise tax share is derived from average cigarette prices (see above) and the tax structure established in the IEPS and Value Added Tax (IVA) laws [40, 41]. A retailer margin of 30% of the price to the retailer was considered. More details on the components of cigarette prices are provided in Supplementary Table A1.

#### Price elasticity

Consistent with the predictions of economic theory, several studies for high-income countries have documented that low-income population is more responsive to price changes [15, 42]. The growing evidence for low- and middle-income countries also points in the same direction [43]. For this study, the average price elasticity and its gradient by income were taken from previous literature reviews [15, 44], although we checked the validity of the former using recent data for Mexico (Supplementary Table A4). The updated estimate was consistent with earlier studies for the country that employed different estimation methods [45]. A global price elasticity of − 0.4 implies a decrease in consumption of 4% with a price increase of 10%, i.e., a price increase of 44% —the modelled scenario— is expected to reduce consumption by 18%. Of this reduction, half is assumed to correspond to quitting among current smokers, and half to the reduction in the cigarettes smoked among those who continue smoking. The beneficial effects of the latter are ignored.

All monetary figures are presented in both Mexican pesos of 2020 (MX$) and international dollars (PPP$) —adjusted for purchasing power parity (PPP). The PPP conversion factor is from the World Bank [37].

## Results

### Smoking patterns at baseline

Smoking patterns vary considerably across regions (Table 1). The prevalence is higher in the Northeast (18% of individuals 10 years or older) but the Centre has the highest number of smokers (5.8 million or 36% of all smokers) due to the high population density. Nearly half of all smokers are adults aged 25 to 44 years (7.5 million). Consumption is also high in the North, although the highest figure corresponds to the West (4.6 cigarettes per day on average).

While smoking exhibits a positive socio-economic gradient overall, there are 5.2 million smokers in the two poorest quintiles that represent 32% of all smokers. Importantly, the opposite pattern is observed in the South, where 60% of the smokers are in the two bottom quintiles (1.4 million). This simply reflects the disproportioned concentration of low-income population in this region. Indeed, the South is the region with the highest poverty incidence: 60.9% are poor and 19.5% are extremely poor, compared to about 28% and less than 3% in the North, and nearly 40% and less than 5% in the West and Centre, respectively. Moreover, GDP per capita, public health spending per capita, and social security coverage —which provides the broadest health insurance coverage— are lower in the South, i.e., the population is less protected against the financial consequences of tobacco attributable diseases.

### Health and economic benefits of a substantial tax increase

A substantial one-peso tax increase, equivalent to a 44% price increase, would have important effects on tobacco consumption and smoking prevalence (Table 2). Based on this scenario, 1.5 million smokers would quit across the five regions, with the bottom income quintile having three times as many quitters as the top quintile (386 vs. 129.5 thousand). The Centre would concentrate the largest number of quitters, accounting for one third of the total (518.4 thousand), but the ratio of quitting in the bottom versus top income quintile in the South would be as high as 19.3.

**Table 2:** Cumulative impact of a one-peso tobacco tax increase on health and financing outcomes per region in Mexico

Outcomes by income group	Regions					Total
	Northwest	Northeast	West	Centre	South	
**Number of smokers who quit smoking (in thousands)**						
First (bottom 20%)	46.7	38.8	45.5	103.7	151.3	386.0
Second	52.3	50.4	78.3	150.3	80.5	411.9
Third	49.4	52.9	75.4	125.0	34.2	336.7
Fourth	33.3	41.5	47.8	88.2	24.1	234.8
Fifth (top 20%)	18.2	26.3	25.8	51.3	7.9	129.5
Total	200.0	209.8	272.8	518.4	297.9	1499.0
First:fifth ratio	2.6	1.5	1.8	2.0	19.3	3.0
**Total life years gained (in thousands)**						
First (bottom 20%)	392.0	321.0	375.9	873.2	1261.6	3223.6
Second	408.2	422.7	676.3	1332.0	703.4	3542.6
Third	398.1	453.0	649.6	1053.8	289.6	2844.1
Fourth	272.1	351.1	405.6	736.9	203.4	1969.1
Fifth (top 20%)	147.0	216.6	216.0	416.8	65.9	1062.2
Total	1617.4	1764.4	2323.3	4412.7	2523.8	12,641.7
First:fifth ratio	2.7	1.5	1.7	2.1	19.1	3.0
**Total deaths averted (in thousands)**						
First (bottom 20%)	19.6	16.2	18.7	43.9	62.9	161.2
Second	20.8	21.1	33.4	65.3	34.6	175.4
Third	20.1	22.5	32.3	52.6	14.5	141.8
Fourth	13.5	17.6	20.2	36.9	10.1	98.4
Fifth (top 20%)	7.4	10.9	11.0	20.8	3.3	53.3
Total	81.4	88.2	115.5	219.6	125.4	630.1
First:fifth ratio	2.6	1.5	1.7	2.1	19.2	3.0
**Treatment cost averted (MX$ (PPP$), in billions)**						
First (bottom 20%)	1.4 (0.1)	1.1 (0.1)	1.3 (0.1)	2.8 (0.3)	3.9 (0.4)	10.5 (1.1)
Second	1.4 (0.1)	1.6 (0.2)	2.4 (0.3)	4.4 (0.5)	2.4 (0.3)	12.2 (1.3)
Third	1.5 (0.2)	1.7 (0.2)	2.5 (0.3)	3.9 (0.4)	1.0 (0.1)	10.6 (1.1)
Fourth	1.0 (0.1)	1.3 (0.1)	1.6 (0.2)	2.8 (0.3)	0.7 (0.1)	7.4 (0.8)
Fifth (top 20%)	0.5 (0.1)	0.8 (0.1)	0.9 (0.1)	1.4 (0.1)	0.2 (0.0)	3.8 (0.4)
Total	5.9 (0.6)	6.4 (0.7)	8.8 (0.9)	15.2 (1.6)	8.3 (0.9)	44.6 (4.6)
First:fifth ratio	2.7	1.4	1.4	2.0	16.4	2.7
**Additional tax revenue per year (MX$ (PPP$), in billions)**						
First (bottom 20%)	0.07 (0.01)	0.05 (0.01)	0.13 (0.01)	0.10 (0.01)	0.11 (0.01)	0.46 (0.05)
Second	0.41 (0.04)	0.20 (0.02)	0.42 (0.05)	0.37 (0.04)	0.11 (0.01)	1.50 (0.16)
Third	0.65 (0.07)	0.47 (0.05)	0.77 (0.08)	0.93 (0.10)	0.20 (0.02)	3.02 (0.33)
Fourth	0.80 (0.09)	0.78 (0.08)	1.29 (0.14)	1.44 (0.16)	0.33 (0.04)	4.63 (0.50)
Fifth (top 20%)	1.07 (0.12)	1.27 (0.14)	1.76 (0.19)	2.22 (0.24)	0.28 (0.03)	6.59 (0.71)
Total	2.99 (0.32)	2.77 (03.30)	4.36 (0.47)	5.06 (0.55)	1.02 (0.11)	16.21 (1.75)
First:fifth ratio	0.07	0.04	0.07	0.05	0.40	0.07
**Number of people avoiding poverty (in thousands)**						
First (bottom 20%)	3.5	2.6	2.9	6.3	5.8	21.0
Second	18.4	20.1	29.6	56.5	32.7	157.3
Third	7.3	6.6	18.2	39.3	11.1	82.4
Fourth	2.1	2.5	2.9	4.9	1.6	14.0
Fifth (top 20%)	0.0	0.0	0.4	0.8	0.2	1.4
Total	31.2	31.8	54.0	107.8	51.3	276.1
First:fifth ratio	–	–	7.3	7.9	29.0	15.0
**Number of people avoiding catastrophic health expenditures (in thousands)**						
First (bottom 20%)	18.2	14.1	15.9	35.8	52.3	136.3
Second	18.4	20.2	29.6	56.5	32.7	157.4
Third	19.3	21.6	31.0	50.5	13.9	136.1
Fourth	12.2	17.3	19.6	35.6	9.7	94.5
Fifth (top 20%)	5.8	8.6	9.9	16.6	3.2	44.1
Total	73.9	81.8	106.1	195.0	111.7	568.4
First:fifth ratio	3.1	1.6	1.6	2.2	16.4	3.1

A one-peso tax increase is roughly equivalent to a 44% increase in price. The benefits of the tax increase would be observed during the lifetime of the current smoking cohort, except for the additional tax revenue that would be annual. Northeast = Baja California, Baja California Sur, Chihuahua, Durango, Sonora and Sinaloa. Northwest = Coahuila, Nuevo León, San Luis Potosí, Tamaulipas and Zacatecas. West = Aguascalientes, Colima, Guanajuato, Jalisco, Michoacán and Nayarit. Centre = Ciudad de México, Estado de México, Hidalgo, Morelos, Puebla, Querétaro and Tlaxcala. South = Campeche, Chiapas, Guerrero, Oaxaca, Quintana Roo, Tabasco, Veracruz and Yucatán. PPP$ = International dollars adjusted for purchasing power parity 2019. MX$ = Mexican pesos of 2020

As a result of quitting, nearly 630 thousand premature deaths would be averted and 12.6 million life years would be gained, of which more than half would correspond to the central (4.4 million) and southern (2.5 million) regions of Mexico. Following the pattern of quitting across quintiles, the bottom income group would gain three times more life years than the top quintile (3.2 vs. 1.1 million life years), and the largest gain for the most deprived would occur in the South (ratio of 19:1).

The tobacco excise tax increase would also bring important economic benefits. Costs averted for treating the four major tobacco attributable diseases would be about MX$44.6 billion (or PPP$4.6 billion), with higher savings in the first three quintiles (MX$33.3 billion or PPP$3.5 billion). Overall, averted costs would be 2.7 times higher in the bottom quintile compared to the top (MX$10.5 vs. 3.8 billion). The largest impacts are observed in regions with more expected deaths averted: savings in the Centre, West and South would amount to MX$15.2 billion (PPP$1.6 billion), MX$8.8 (PPP$0.9 billion), and MX$8.3 billion (PPP$0.9 billion), respectively.

In contrast, the additional tax revenue from the top income quintile would be 14 times that from the bottom quintile (MX$6.59 or PPP$0.71 billion vs. MX$0.46 or PPP$0.05 billion). Moreover, this would imply an increase of the top vs. bottom income quintile ratio from 9 to 14. Nearly 58% of the additional tax revenue would be from the Centre and West (MX$9.4 billion), as these regions have the highest record of (after policy) smokers and smoking intensity, respectively. The tax increase would yield an additional revenue of MX$16.21 (PPP$1.75) billion per year, and a total annual revenue of MX$59.9 billion that represent 0.3% of GDP and 9.4% of public health expenditure in Mexico approximately. Total yearly consumption would fall from 1397 million packs at baseline to 1151 million packs, i.e., 17.6%.

The treatment costs averted also imply that 276 thousand individuals would avoid falling below the poverty line, including 21 thousand in the bottom income group and 157 thousand in the second lowest income group. Likewise, over half million individuals (568.4 thousand) would avoid catastrophic health expenditures, half of them from the two bottom income groups (136.3 and 157.4 thousand in the first and second quintile, respectively). Again, the largest benefits are observed in the Centre (with 107.8 thousand individuals avoiding poverty and 195 thousand avoiding catastrophic health expenditures) and the South (with 51.3 thousand individuals avoiding poverty and 111.7 thousand avoiding catastrophic health expenditures).

### Sensitivity analyses

**Fig. 2:**
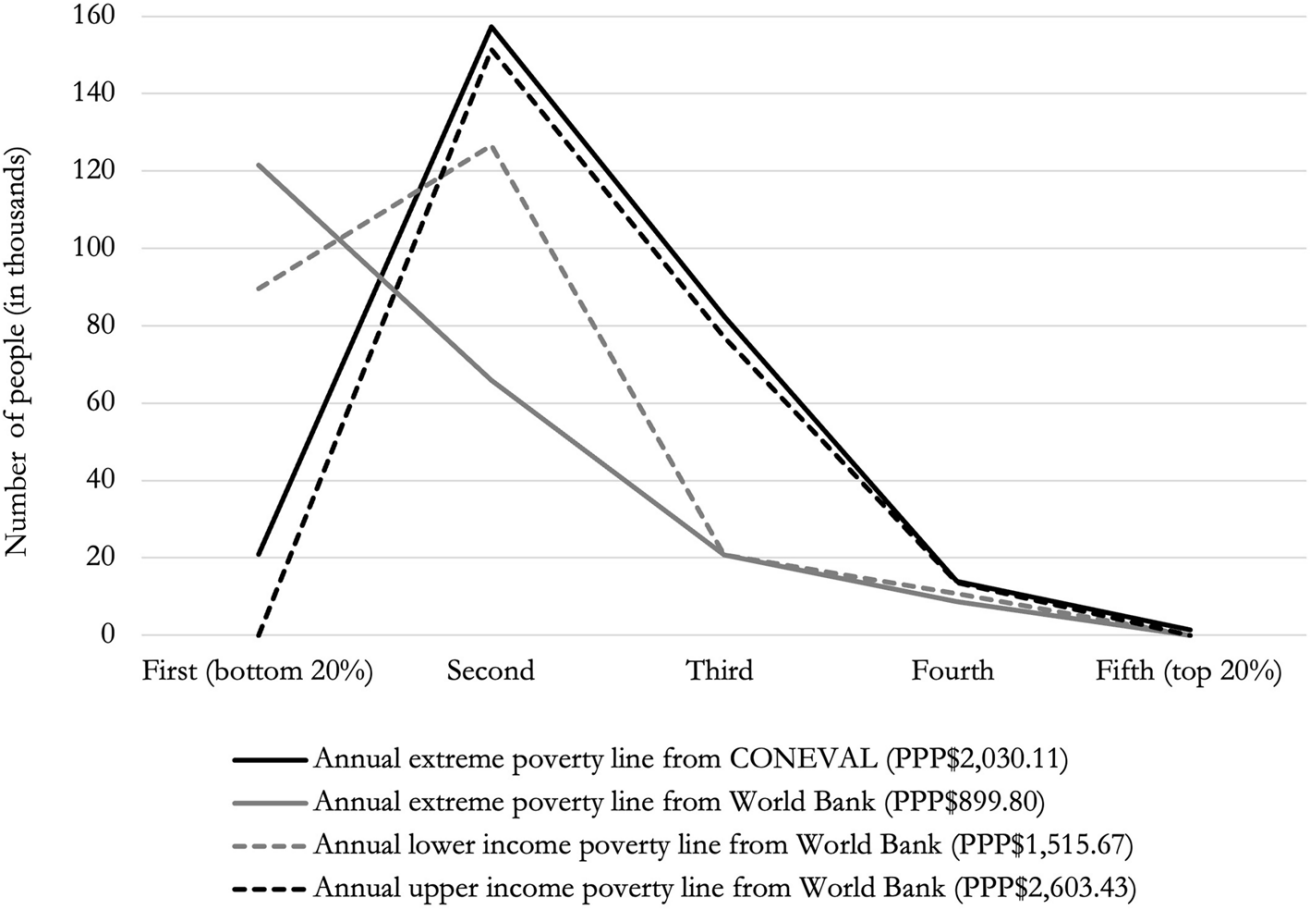
Number of people averting poverty by income quintile after a one-peso tax increase (in thousands). PPP$ = International dollars adjusted for purchasing power parity 2018. The annual extreme poverty line from CONEVAL corresponds to the value of the food basket per person in urban areas

Figure 2 further explores averted poverty estimates. In particular, we compare the results obtained with the official poverty line to those obtained using international poverty lines. The most salient difference is observed when the extreme international poverty line is employed. Since this line is considerably lower, the resulting poverty headcount ratio in the bottom quintile at baseline is the lowest and thus the estimated capacity of the tax to prevent people from falling into poverty is the greatest. Indeed, if we use this poverty line there is an inverse relation between income group and the number of people avoiding poverty. Conversely, if we consider the highest poverty line (the upper income poverty line from the World Bank), the entire population of the bottom quintile is classified as poor at baseline and thus the number of poor avoided by the tax is zero. In this case, the largest number of poor averted corresponds to the second lowest quintile, leading to the inverted v-shaped curve that is also observed with the official extreme urban poverty line. Two important conclusions can be drawn from this analysis. First, the choice of poverty line importantly affects the estimates of poor avoided. While international poverty lines facilitate between-country comparisons, national poverty lines for country-specific estimates —if available— could yield more policy relevant results at the local level. In this study, both poverty lines and income data correspond to those employed to obtain the official headcount poverty ratio at baseline. Second, this indicator does not capture the whole concept of impoverishing expenditure. Treatment costs of tobacco attributable diseases not only push some households into poverty, but also push poor households further into poverty. This consideration is particularly relevant in low- and middle-income countries, where most of the bottom income quintile is often already in poverty. Hence, tobacco taxes prevent new poverty cases and further impoverishment; the poor averted indicator only considers the former.

Since Mexico is carrying out a major health system reform, we also consider an alternative scenario in which the uninsured have the same financial protection as SP beneficiaries. As INSABI no longer requires affiliation, all services previously offered to SP beneficiaries are, in principle, currently available to the population without social security. This implies that they should also have covered the full COPD treatment, while progressive fees would apply for the other three conditions. Because this scenario does not modify the number of smokers who quit, the estimates of deaths averted, years of life gained, and total costs averted remain unchanged. However, the distribution of the costs between the insurer and the insured would be different. In the main scenario, 66% of the total costs averted are borne by the State, while 34% are out-of-pocket (OOP) expenses. On the other hand, in this alternative scenario little more than three-quarters would be borne by the State. Therefore, the number of individuals avoiding poverty and catastrophic health expenditures would drop to two-thirds of those estimated for the main scenario, although a similar gradient would be observed (data not shown but available on request). In sum, given that under this scenario most of the treatment costs are covered by the public health system, the State would obtain a relatively greater proportion of the benefits derived from the costs avoided due to the tax increase.

The third sensitivity analysis considers a slightly higher tax increase of 1.15 pesos that corresponds to a 50% price increase (Supplementary Table A5). In general, the first to fifth quintile ratio is similar for both health and economic outcomes, although the gains in absolute terms are naturally higher. We also show the effect of varying the price elasticity (Supplementary Table A6 and Fig. A1). The first scenario uses a slightly higher average price elasticity that comes from estimates based on recent data for Mexico (−0.479; see Supplementary Table A4). Since the elasticity of the income groups at the extremes remains unchanged, this scenario translates into an increase in benefits for the third quintile. The second scenario uses the same average elasticity as the main estimates but assumes a smaller difference between quintiles. This translates into a reduction in the first to fifth quintile ratio for all outcomes, although gains in the first quintile remain at least close to and more commonly above those of the fifth quintile, especially in the South. Therefore, the distribution of the benefits from tobacco taxes will depend on their differential effect on prevalence, as measured by price elasticities, and the distribution of smokers across quintiles. In southern Mexico, the ratio of quitting smoking in the bottom versus top income group remains high in the second scenario that assumes a less steep gradient because smokers are more concentrated in the first quintile than the fifth in this region.

Finally, we compare the totals in Table 2 that correspond to the sum of the regional estimates to the results that are obtained by running the model with national level inputs (Supplementary Table A3) to check for internal consistency of the model. Supplementary Table A7 shows that health outcomes are identical in both cases, and only slight differences are observed in financial indicators. These differences are mainly driven by differences in utilisation rates and shares of tobacco attributable diseases between regions, which affect the estimation of costs averted and, therefore, that of the other financial indicators. However, given that the objective was to take advantage of the information available at the subnational level, and considering that the differences were relatively small (particularly in the first to fifth ratio), no adjustments to the main results were made.

## Discussion

The results of this study confirm that tobacco tax increases are associated with important pro-poor health and financial benefits. In particular, a one-peso tax increase per cigarette could reduce smoking prevalence in Mexico by 9.3% —equivalent to about 1.5 million fewer smokers—, and this decrease would be more significant for the lowest two quintiles, where reductions would amount to 17 and 14%, respectively. As a result, 630 thousand deaths would be averted nationwide among the current smoking cohort, 26% in the lowest quintile compared to 9% in the highest quintile (Supplementary Fig. A2). This positive impact on health is also transferred to economic indicators, resulting in meaningful savings for the health system, the smokers and their households (44.6 billion pesos in total). Moreover, about 276 thousand individuals, mostly from the two bottom quintiles (65%), would avoid falling below the poverty line. At the same time, this policy would generate an additional revenue of 16 billion pesos per year, which would be borne mainly (69%) by the two top quintiles.

The distributional effects of tobacco taxation are consistent across regions: the most deprived are generally more benefited than the most affluent. However, the relative benefits for the bottom quintile would be notably higher in the South, since most of the poor smokers are concentrated in this region (39% of all smokers in the first quintile). Specifically, health gains (both deaths averted and life years gained) would be 19 times higher for the bottom quintile compared to the top in the South. In absolute terms, the largest gains would be observed in the Centre, the most densely populated region, e.g., it is estimated that slightly more than half a million smokers or 8.9% of baseline smokers would quit. Yet, those expected to quit in the Northeast, Northwest and West would also represent between 8.3 and 8.9% of current smokers, bringing significant benefits to these regions as well. In general, subnational estimates highlight important benefits at the local level that would otherwise go unnoticed and thus may be key to engaging local decision makers in national discussions to advance stronger tobacco taxation measures. The subnational variability described in this study has also been measured for India and is very likely present in other countries such as Brazil, where recent reductions in smoking prevalence have been heterogeneous across states [46, 47].

For several decades, national health and tobacco surveys have revealed the tobacco epidemic exhibits great variability among different regions in Mexico [18, 48–51], with a higher prevalence in the North and more significant number of smokers in the central and western regions. Likewise, it is known that the southern region has the lowest prevalence of consumption but also faces the most significant challenges in terms of health infrastructure to address the burden of disease attributable to this risk factor. The findings from this subnational analysis are broadly consistent with previous data.

Unlike a previous ECEA that focused on male, daily smokers [16], we also considered female and occasional smokers since they constitute an important share of all cigarette smokers in Mexico (28 and 57% in the case of female and occasional smokers, respectively) and should therefore be included to provide a more complete picture of the overall effects of tobacco taxation and to understand any potential gendered impacts of tobacco taxation strategies. Additionally, this allowed increasing the sample size to estimate the number of smokers by quintile and age group directly from ENSANUT, which was key to improve the bottom-up calibration of the model (regional to national), and to better portray the actual distribution of smokers in each region. We also calibrated the model to obtain consistent estimates of tax revenue, given that we had reliable information on the actual figure for the baseline year. Frequently, consumption is underreported in smoker surveys and the case of ENSANUT does not seem to be the exception. For the adjustment, we assumed that it is the number of cigarettes consumed (particularly among occasional smokers) and not the number of smokers that is underreported. Indeed, previous validations of self-reported smoking with biological markers suggest that underreporting among adolescents does not exceed 18%, although this population group tends to be the one in which this problem is higher [52]. Total consumption figures after this calibration procedure (1400 million cigarette packs at baseline) were consistent with other estimates for the country in recent years [25].

The study presents a number of limitations. First, we employed a static model with a focus on the current cohort of smokers, thus we do not take into account important long-term benefits of tobacco taxation such as those derived from the reduction of smoking initiation. Second, some inputs were not available at the subnational level, although these represent a small share of the total number of inputs. Future research should consider the analysis of geographic and socioeconomic heterogeneity in key parameters so that this and similar simulation models can make more precise disaggregated projections of tobacco tax effects. Although much progress has been made in studying price elasticities in low- and middle-income countries [43], including in Mexico, estimates for different population groups remain scarce. Something similar can be said about the costs of illness. In the case of Mexico, there are very detailed costs analyses for different types of providers, but regional heterogeneity has been little studied. Although our estimates of costs avoided due to the tax increase consider inequalities in health care access across income groups and regions, they do not capture possible differences in average costs between regions. Third, although the evidence suggests that it is reasonable to assume that half of current smokers will die from tobacco attributable diseases [16, 30], attributable mortality estimates might be less accurate than those from more complex models that follow a hypothetical cohort for which they calculate individual annual risks of disease incidence, disease progression and death [6, 21]. The main contribution of this model, however, lies in that it provides a better understanding of the distribution of tobacco attributable deaths and, therefore, the distribution of tax benefits across income groups and regions using a relatively simple specification. Moreover, the model links fiscal policy strategies for health to economic and poverty outcomes: it shows that tobacco taxation is not only critically important to curb tobacco use but it also stands to avert poverty due to health care expenditure, particularly among those most impacted and least able to pay.

It is essential that Mexico continues strengthening tobacco taxation in line with the levels recommended in the FCTC. While important steps were taken at the end of 2019 to break out the nearly decade-long stagnation (see the introduction), a substantial tax increase is required to reduce by one third premature mortality from noncommunicable diseases —a goal of the 2030 Agenda [53]. Furthermore, the findings of this study show that tobacco taxes can also contribute to other sustainable development goals such as poverty reduction —because they reduce poverty cases associated with treatment costs of tobacco attributable diseases— and universal health coverage —if tax revenue is invested, at least in part, in the health system.

## Conclusions

Tobacco taxation remains the single, most cost-effective public policy to deter the smoking epidemic. This analysis confirms that a substantial cigarette price increase would reduce smoking prevalence, and this reduction would mainly occur among the most deprived population. Consequently, the most significant health benefits —mortality avoided and years of life gained— would be higher in the lowest quintiles, especially in the South, the most impoverished region of Mexico. These benefits would also translate into economic terms: treatment costs avoided, such as out-of-pocket expenses, would significantly prevent many smokers from falling below the poverty line and facing unmanageable health care costs.

This study provides for the first time estimates of the health and economic benefits of tobacco fiscal policy at the subnational level in Mexico, which may be key to strengthen local actions, as well as to promote a more equitable allocation of health resources within and between regions.

## Supplementary Information

**Additional file 1.**

## Data Availability

Most data analysed during the study are included in this article and its supplementary information files (Supplementary Table A3). Full datasets used are available from the corresponding author on reasonable request.
