# Supplementary material for: The distributional effects of tobacco tax increases across regions in Mexico: an extended cost-effectiveness analysis
Source: Int J Equity Health. 2022 Jan 20;21:8. doi: 10.1186/s12939-021-01603-2 (PMC8772114; doi:10.1186/s12939-021-01603-2)
Supplement: Supplementary file 1 — Additional file 1. [file 12939_2021_1603_MOESM1_ESM.docx]

**Additional File 1**

**Table A1. Cigarette price structure, Mexico 2020**

|  |  |  | **Baseline year 2020** | | **One-peso tobacco tax increase** | |
| --- | --- | --- | --- | --- | --- | --- |
|  |  |  | **MX$2020** | **As % of the price to the consumer** | **MX$2021** | **As % of the price to the consumer** |
|  |  |  | **(1)** | **(2)** | **(3)** | **(4)** |
| A | Price to the consumer per 20-cigarette pack | | 56.43 | 100.0% | 81.21 | 100.0% |
| B | Value Added Tax (VAT) | | 7.78 | 13.8% | 11.20 | 13.8% |
| C | Retailer margin | | 4.01 | 7.1% | 4.15 | 5.1% |
|  | Excise tax (IEPS) | |  |  |  |  |
| D |  | Ad valorem excise tax | 21.39 | 37.9% | 22.13 | 27.3% |
| E |  | Specific excise tax | 9.89 | 17.5% | 29.89 | 36.8% |
| F | Price to the retailer per pack before taxes | | 13.37 | 23.7% | 13.83 | 17.0% |

Notes:

*Estimates at baseline (columns 1-2).* The price to the consumer (A) is a weighted average of cigarette prices per 20-cigarette packs from INEGI; market shares by brand were employed as weights (Gutiérrez et al. 2020). The IVA (B) is 16% of the price to the retailer. The retailer margin (C) is 30% of the price to the retailer. The ad valorem IEPS (D) is 160% of the price to the retailer, and the specific IEPS (E) is 0.4944 pesos per cigarette.

*Estimates after one-peso tax increase (columns 3-4).* Estimates under the simulated scenario consider that the price to the retailer (F) is adjusted by annual inflation (3.5%). A to E under this scenario are based on F, with E = 1.4944 pesos per cigarette.

**Table A2. Calibrated average cigarette consumption per day by income group**

| **Income group** | **Northwest** | **Northeast** | **West** | **Centre** | **South** | **National** |
| --- | --- | --- | --- | --- | --- | --- |
| Q1 | 5.9 | 6.5 | 6.7 | 2.8 | 2.3 | 3.8 |
| Q2 | 7.3 | 4.5 | 6.7 | 3.2 | 1.8 | 4.3 |
| Q3 | 6.6 | 5.0 | 6.2 | 4.1 | 3.1 | 5.0 |
| Q4 | 6.0 | 5.1 | 7.0 | 4.4 | 3.5 | 5.1 |
| Q5 | 5.7 | 4.8 | 7.0 | 4.3 | 3.4 | 5.1 |

Notes: The formulae to obtain the total revenue collected at baseline (t0) and after the tax increase (t1) are the following:

$${Total\_rev}_{t0,q,r}=\mathrm{Smk}_{t0,q,r}\left( 365.25\frac{\mathrm{Cig}_{t0,q,r}}{20} \right)\mathrm{Tax}_{t0}$$

$${Total\_rev}_{t1,q,r}=\mathrm{Smk}_{t1,q,r}\left( 365.25\frac{\mathrm{Cig}_{t0,q,r}}{20} \right)\mathrm{Tax}_{t1}$$

where $\mathrm{Smk}_{t0,q,r}$ is the number of smokers at baseline, in quintile q, region r; $\mathrm{Cig}_{t0,q,r}$ is the average number of cigarettes consumed by smokers per day at baseline, in quintile q, region r; and $\mathrm{Tax}_{t0}$ is the cigarette per pack at baseline. The marginal tax revenue (${\mathrm{Marginal}\_rev}_{q,r}$) is therefore:

$${\mathrm{Marginal}\_rev}_{q,r}={Total\_rev}_{t1,q,r}-{Total\_rev}_{t0,q,r}$$

The total tax revenue at baseline, however, was calibrated to match actual revenue using the following formula:

$${Total\_rev}_{t0\mathrm{cal},q,r}=\left( \frac{{Total\_rev}_{t0,q,r}}{{Total\_rev}_{t0,r}} \right){Total\_rev}_{t0\mathrm{real},r}$$

where ${Total\_rev}_{t0\mathrm{real},r}$ is the real total revenue observed at baseline. Therefore, the calibrated average cigarette consumption per day at t0, shown in the table above, is:

$$\mathrm{Cig}_{t0\mathrm{cal},q,r}=20\left( \frac{{Total\_rev}_{t0\mathrm{cal},q,r}}{365.25*\mathrm{Tax}_{t0}*\mathrm{Smk}_{t0,q,r}} \right)$$

All the parameters used in these formulae and data sources are in Supplementary Table A3. All the model formulae can be found in GTEC (2018).

**Table A3. Model inputs**

| **Variables** | | **National** | **Regional** | | | | |
| --- | --- | --- | --- | --- | --- | --- | --- |
|  |  |  | **Northeast** | **Northwest** | **West** | **Centre** | **South** |
| **SPECIFIC PER REGION** | | | | | | | |
| **Number of smokers by age group at baseline (in thousands)^a,*^** | | | | | | | |
|  | 10-14 | 81.07 | 10.53 | 7.06 | 18.24 | 33.69 | 11.54 |
|  | 15-24 | 3,437.88 | 563.37 | 396.41 | 696.79 | 1,253.28 | 528.03 |
|  | 25-44 | 7,510.69 | 1,132.09 | 1,017.57 | 1,418.59 | 2,761.98 | 1,180.47 |
|  | 45-64 | 3,954.30 | 651.40 | 647.56 | 755.26 | 1,370.34 | 529.74 |
|  | 65-84 | 1,045.55 | 157.64 | 203.34 | 159.06 | 377.16 | 148.35 |
|  | 85+ | 88.91 | 16.19 | 12.15 | 9.21 | 36.95 | 14.42 |
|  | Total | 16,118.40 | 2,531.22 | 2,284.09 | 3,057.16 | 5,833.39 | 2,412.55 |
| **Number of smokers by income group at baseline (in thousands)^a,*^** | | | | | | | |
|  | Q1 | 2,245.03 | 214.15 | 265.96 | 277.25 | 608.31 | 879.36 |
|  | Q2 | 2,952.19 | 366.04 | 408.53 | 556.01 | 1,058.68 | 562.95 |
|  | Q3 | 3,301.24 | 516.54 | 506.02 | 706.91 | 1,225.73 | 346.03 |
|  | Q4 | 3,528.94 | 610.40 | 518.07 | 726.24 | 1,307.04 | 367.20 |
|  | Q5 | 4,090.99 | 824.09 | 585.51 | 790.75 | 1,633.64 | 257.02 |
|  | Total | 16,118.40 | 2,531.22 | 2,284.09 | 3,057.16 | 5,833.39 | 2,412.55 |
| **Average cigarette consumption per day by income group^a^** | | | | | | | |
|  | Q1 | 2.6 | 4.4 | 4.0 | 4.6 | 1.9 | 1.6 |
|  | Q2 | 2.9 | 3.1 | 5.0 | 4.6 | 2.2 | 1.2 |
|  | Q3 | 3.4 | 3.4 | 4.5 | 4.2 | 2.8 | 2.1 |
|  | Q4 | 3.5 | 3.5 | 4.1 | 4.8 | 3.0 | 2.4 |
|  | Q5 | 3.5 | 3.3 | 3.9 | 4.8 | 2.9 | 2.3 |
| **Main tobacco attributable diseases as a share of total deaths^b^** | | | | | | | |
|  | COPD | 33% | 29% | 28% | 39% | 34% | 31% |
|  | Stroke | 11% | 10% | 9% | 9% | 11% | 12% |
|  | Heart disease | 42% | 43% | 44% | 37% | 42% | 44% |
|  | Lung cancer | 15% | 17% | 20% | 15% | 14% | 13% |
| **Health utilization, relative to Q3^a^** | | | | | | | |
|  | Q1 | 0.86 | 0.91 | 0.97 | 0.89 | 0.85 | 0.87 |
|  | Q2 | 0.94 | 1.00 | 0.92 | 0.92 | 0.90 | 0.98 |
|  | Q3 | 1.00 | 1.00 | 1.00 | 1.00 | 1.00 | 1.00 |
|  | Q4 | 1.00 | 1.02 | 0.94 | 1.01 | 1.00 | 1.00 |
|  | Q5 | 0.96 | 0.97 | 0.96 | 1.05 | 0.90 | 1.01 |
| **Insurance coverage rate^a^** | | | | | | | |
| Rural | |  |  |  |  |  |  |
|  | Social security | 20.1% | 25.5% | 35.4% | 28.0% | 18.2% | 12.7% |
|  | Seguro Popular | 65.0% | 62.6% | 50.3% | 55.4% | 67.1% | 72.4% |
|  | None | 14.0% | 12.0% | 14.3% | 16.5% | 14.7% | 14.9% |
| Urban | |  |  |  |  |  |  |
|  | Social security | 52.6% | 66.1% | 64.6% | 52.6% | 47.9% | 41.7% |
|  | Seguro Popular | 27.7% | 19.9% | 18.9% | 28.9% | 28.0% | 39.1% |
|  | None | 19.0% | 14.0% | 16.5% | 18.5% | 24.1% | 19.3% |
| **Average annual per capital household income by income group, (MX$ (PPP$), 2018)^c^** | | | | | | | |
|  | Q1 | 13,399.89 | 14,387.62 | 14,113.67 | 14,570.22 | 14,614.20 | 12,030.33 |
|  |  | (1,468.16) | (1,576.38) | (1,546.37) | (1,596.39) | (1,601.21) | (1,318.10) |
|  | Q2 | 25,619.53 | 25,903.74 | 25,820.62 | 25,818.68 | 25,582.61 | 25,300.34 |
|  |  | (2,807.01) | (2,838.14) | (2,829.04) | (2,828.82) | (2,802.96) | (2,772.03) |
|  | Q3 | 37,225.60 | 37,446.38 | 37,322.28 | 37,160.70 | 37,280.42 | 36,936.44 |
|  |  | (4,078.62) | (4,102.81) | (4,089.22) | (4,071.51) | (4,084.63) | (4,046.94) |
|  | Q4 | 54,985.54 | 55,174.82 | 55,021.79 | 54,915.65 | 54,976.40 | 54,870.34 |
|  |  | (6,024.49) | (6,045.23) | (6,028.46) | (6,016.84) | (6,023.49) | (6,011.87) |
|  | Q5 | 139,816.24 | 135,206.74 | 42,980.39 | 133,554.84 | 149,087.99 | 128,235.26 |
|  |  | (15,318.97) | (14,813.93) | (15,665.65) | (14,632.94) | (16,334.83) | (14,050.10) |
|  | Total | 54,223.31 | 65,393.87 | 61,988.57 | 56,547.88 | 56,744.64 | 37,830.97 |
|  |  | (5,940.98) | (7,164.88) | (6,791.78) | (6,195.67) | (6,217.23) | (4,144.95) |
|  | | | | | | | |
| **HOMOGENEOUS ACROSS REGIONS** | | | | | | | |
| **Annual treatment cost from tobacco attributable diseases, (MX$ (PPP$), 2018)^d^** | | | | | | | |
|  | COPD | 103,234.68 |  |  |  |  |  |
|  |  | (11,310.91) |  |  |  |  |  |
|  | Stroke | 35,287.81 |  |  |  |  |  |
|  |  | (3,866.31) |  |  |  |  |  |
|  | Heart disease | 41,540.99 |  |  |  |  |  |
|  |  | (4,551.44) |  |  |  |  |  |
|  | Lung cancer | 118,169.73 |  |  |  |  |  |
|  |  | (12,947.27) |  |  |  |  |  |
| **Probability of seeking health care^d^** | | | | | | | |
|  | COPD | 0.96 |  |  |  |  |  |
|  | Stroke | 0.96 |  |  |  |  |  |
|  | Heart disease | 0.96 |  |  |  |  |  |
|  | Lung cancer | 0.96 |  |  |  |  |  |
| **Risk reduction by age group^d^** | | | | | | | |
|  | 10-14 | 1 |  |  |  |  |  |
|  | 15-24 | 0.97 |  |  |  |  |  |
|  | 25-44 | 0.85 |  |  |  |  |  |
|  | 45-64 | 0.75 |  |  |  |  |  |
|  | 65-84 | 0.25 |  |  |  |  |  |
|  | 85+ | 0.02 |  |  |  |  |  |
| **Financial protection (insurance coverage of treatment costs)^e^** | | | | | | | |
|  | Social security | 100% |  |  |  |  |  |
|  | Seguro Popular |  |  |  |  |  |  |
|  | COPD | 100% |  |  |  |  |  |
|  | Stroke, heart disease and lung cancer | | | | | | |
|  | Q1 | 32.0% |  |  |  |  |  |
|  | Q2 | 24.0% |  |  |  |  |  |
|  | Q3 | 17.3% |  |  |  |  |  |
|  | Q4 | 7.2% |  |  |  |  |  |
|  | Q5 | 2.4% |  |  |  |  |  |
|  | Uninsured | 0.0% |  |  |  |  |  |
| **Annual poverty line, Mexican pesos, (MX$ (PPP$), 2018)^f^** | | | | | | | |
|  |  | 18,528.84 |  |  |  |  |  |
|  |  | (2,030.11) |  |  |  |  |  |
| **Average price per 20 cigarette pack, (MX$2020, (PPP$2019))^g^** | | | | | | | |
|  |  | 56.43 (6.08) |  |  |  |  |  |
| **Price elasticity by income group^d^** | | | | | | | |
|  | Q1 | -0.64 |  |  |  |  |  |
|  | Q2 | -0.51 |  |  |  |  |  |
|  | Q3 | -0.38 |  |  |  |  |  |
|  | Q4 | -0.25 |  |  |  |  |  |
|  | Q5 | -0.12 |  |  |  |  |  |
| **Specific excise tax (baseline / after tax increase)^h^** | | | | | | | |
|  |  | 0.4944 / 1.4944 | |  |  |  |  |
| **PPP conversion factor (2018 / 2019)^i^** | | | | | | | |
|  |  | 9.127 / 9.278 |  |  |  |  |  |

Sources: ^a^Instituto Nacional de Estadística y Geografía (INEGI), Instituto Nacional de Salud Pública (INSP). *Encuesta Nacional de Salud y Nutrición (ENSANUT) 2018-19*. Available at: https://ensanut.insp.mx.

^b^Global Burden of Disease Collaborative Network. *Global Burden of Disease Study 2017 (GBD 2017) Results*. Seattle: Institute for Health Metrics and Evaluation (IHME); 2018.

^c^Instituto Nacional de Estadística y Geografía (INEGI). *Encuesta Nacional de Estadística y Geografía (ENIGH)*. Aguascalientes: INEGI. Available at: https://www.inegi.org.mx/programas/enigh/nc/2018/

^d^Global Tobacco Economics Consortium. The health, poverty, and financial consequences of a cigarette price increase among 500 million male smokers in 13 middle income countries: compartmental model study. *BMJ* 2018;361:k1162. doi:10.1136/bmj.k1162.

^e^Insurance schemes regulations; Seguro Popular figures by quintiles for diseases other than COPD from Arrieta O, Quintana-Carrillo RH, Ahumada-Curiel G, Corona-Cruz JF, Correa-Acevedo E, et al. Medical care costs incurred by patients with smoking-related non-small cell lung cáncer treated at the National Cancer Institute of Mexico, *Tobacco Induced Diseases* 2014;12:25.

^f^Consejo Nacional de Evaluación de la Política de Desarrollo Social (CONEVAL). *Líneas de ingreso*. Mexico City: CONEVAL. Available at: https://www.coneval.org.mx/Medicion/MP/Paginas/Pobreza_2020.aspx

^g^Prices per pack per brand from INEGI; market share by brand from Gutiérrez-Torres DS, Saenz de Miera Juarez B, Reynales-Shigematsu LM, et al. Trends in cigarette brand preference among Mexican smokers: the rise of Pall Mall. *Tob Control* 2020;0:1-7.

^h^Excise tax law and simulated scenario.

^i^World Bank. World Development Indicators (WDI). Washington, DC: World Bank.

Notes: Northeast = Baja California, Baja California Sur, Chihuahua, Durango, Sonora and Sinaloa. Northwest = Coahuila, Nuevo León, San Luis Potosí, Tamaulipas and Zacatecas. West = Aguascalientes, Colima, Guanajuato, Jalisco, Michoacán and Nayarit. Centre = Ciudad de México, Estado de México, Hidalgo, Morelos, Puebla, Querétaro and Tlaxcala. South = Campeche, Chiapas, Guerrero, Oaxaca, Quintana Roo, Tabasco, Veracruz and Yucatán. *Full data on the number of smokers by age group and income quantile are available upon reasonable request from the corresponding author.

**Table A4. Updated price elasticity for cigarettes in Mexico**

|  | **Clusters = municipalities** | **Clusters = Primary sampling units** |
| --- | --- | --- |
|  | **2016-2018** | **2016-2018** |
| Own price elasticity | -0.4792 | -0.5766 |
| 95% Confidence intervals | (-0.3004,-0.6580) | (-0.5234,-0.6298) |
| Clusters | 1,226 | 5,205 |
| Observations | 7,728 | 7,728 |

Source: Estimates based on pooled data from the National Survey of Household Income and Expenditure (ENIGH) 2016 and 2018 (INEGI).

Notes: Households with zero consumption were excluded from the analysis (n=7,728), i.e., estimates are conditional on smoking (Rijo 2008). Since the ENIGH does not report price information, quarterly cigarette expenditure was divided by the number of cigarettes consumed during the same period to calculate unit values per household. The average unit value was 58.9 pesos per pack of 20 cigarettes. Unit values are endogenous, however, as they reflect the quality of the product. Therefore, we followed the procedure developed by Deaton (1997), the Almost Ideal Demand System (AIDS), that yields consistent estimates of price elasticities based on unit values. Detailed Stata codes to conduct these estimates using household surveys such as ENIGH are available in Deaton (2018). Clusters to capture spatial variation in prices were defined based on municipalities. Control variables for unit values and budget shares regressions included household head characteristics (sex, log of age, and education), and household composition indicators (log of household size, proportion of males, and proportion of adults). All monetary variables were deflated to prices of August 2018. Alternative specifications of clusters based on primary sampling units yield slightly higher elasticity estimates (-0.5766, 95%CI: -0.5234, -0.6298). Price elasticities from previous studies that employed two-part models are within the range of those presented here (Jiménez et al. 2008). Estimates were not disaggregated by income groups (quintiles), as the sample size was insufficient to detect statistically significant differences.

**Table A5. Alternative scenario considering a 1.15 pesos excise tax increase equivalent to a 50% price increase**

| **Outcomes by income group** |  |  | **Tobacco tax increase of 1 peso**  **(A)** | **Tobacco tax increase of 1.15 pesos**  **(B)** |
| --- | --- | --- | --- | --- |
| **Number of smokes who quit smoking (in thousands)** | | | | |
| First (bottom 20%) |  |  | 386.0 | 440.2 |
| Second |  |  | 411.9 | 469.7 |
| Third |  |  | 336.7 | 384.0 |
| Fourth |  |  | 234.8 | 267.8 |
| Fifth (top 20%) |  |  | 129.5 | 147.7 |
| Total |  |  | 1,499.0 | 1,709.5 |
| First:fifth ratio |  |  | 3.0 | 3.0 |
| **Total life years gained (in thousands)** | | | | |
| First (bottom 20%) |  |  | 3,223.6 | 3,676.5 |
| Second |  |  | 3,542.6 | 4,040.2 |
| Third |  |  | 2,844.1 | 3,243.6 |
| Fourth |  |  | 1,969.1 | 2,245.7 |
| Fifth (top 20%) |  |  | 1,062.2 | 1,211.4 |
| Total |  |  | 12,641.7 | 14,417.5 |
| First:fifth ratio |  |  | 3.0 | 3.0 |
| **Total deaths averted (in thousands)** | | | | |
| First (bottom 20%) |  |  | 161.2 | 183.9 |
| Second |  |  | 175.4 | 200.0 |
| Third |  |  | 141.8 | 161.7 |
| Fourth |  |  | 98.4 | 112.2 |
| Fifth (top 20%) |  |  | 53.3 | 60.8 |
| Total |  |  | 630.1 | 718.6 |
| First:fifth ratio |  |  | 3.0 | 3.0 |
| **Treatment cost averted (MX$ (PPP$), in billions)** | | | | |
| First (bottom 20%) |  |  | 10.5 (1.1) | 11.9 (1.2) |
| Second |  |  | 12.2 (1.3) | 14.0 (1.5) |
| Third |  |  | 10.6 (1.1) | 12.1 (1.3) |
| Fourth |  |  | 7.4 (0.8) | 8.4 (0.9) |
| Fifth (top 20%) |  |  | 3.8 (0.4) | 4.4 (0.5) |
| Total |  |  | 44.6 (4.6) | 50.7 (5.3) |
| First:fifth ratio |  |  | 2.7 | 2.7 |
| **Additional tax revenues per year (MX$ (PPP$), in billions)** | | | | |
| First (bottom 20%) |  |  | 0.46 (0.05) | 0.36 (0.04) |
| Second |  |  | 1.50 (0.16) | 1.44 (0.16) |
| Third |  |  | 3.02 (0.33) | 3.29 (0.35) |
| Fourth |  |  | 4.63 (0.50) | 5.09 (0.55) |
| Fifth (top 20%) |  |  | 6.59 (0.71) | 7.59 (0.82) |
| Total |  |  | 16.21 (1.75) | 17.78 (1.92) |
| First:fifth ratio |  |  | 0.07 | 0.05 |
| **Number of people averting poverty (in thousands)** | | | | |
| First (bottom 20%) |  |  | 21.0 | 23.5 |
| Second |  |  | 157.3 | 180.0 |
| Third |  |  | 82.4 | 99.2 |
| Fourth |  |  | 14.0 | 16.4 |
| Fifth (top 20%) |  |  | 1.4 | 2.3 |
| Total |  |  | 276.1 | 321.3 |
| First:fifth ratio |  |  | 15.0 | 10.4 |
| **Number of people avoiding catastrophic health expenditures (in thousands)** | | | | |
| First (bottom 20%) |  |  | 136.3 | 152.5 |
| Second |  |  | 157.4 | 180.0 |
| Third |  |  | 136.1 | 155.2 |
| Fourth |  |  | 94.5 | 107.8 |
| Fifth (top 20%) |  |  | 44.1 | 51.3 |
| Total |  |  | 568.4 | 646.8 |
| First:fifth ratio |  |  | 3.1 | 3.0 |

Notes: The modelled scenario considers a 1.15 pesos tobacco tax increase (from 0.4944 pesos per cigarette to 1.6444), equivalent to a 50% price increase. The benefits of the tax increase would be observed during the lifetime of the current smoking cohort, except for the additional tax revenue that would be annual. PPP$ = International dollars adjusted for purchasing power parity 2019. MX$ = Mexican pesos of 2020. Estimates under the one-peso scenario (A) correspond to the last column (Total) of Table 2.

**Table A6. Sensitivity analysis for health and financial outcomes by varying price elasticities**

| **Outcomes by income group** | **Scenario 1: Elasticity for quintile 3 of -0.4792** | | | | | **Scenario 2: Lower gradient elasticity** | | | | |
| --- | --- | --- | --- | --- | --- | --- | --- | --- | --- | --- |
|  | **Northwest** | **Northeast** | **West** | **Center** | **South** | **Northwest** | **Northeast** | **West** | **Center** | **South** |
| **Number of smokers who quit smoking (in thousands)** | | | | | | | | | | |
| First (bottom 20%) | 46.7 | 38.8 | 45.5 | 103.7 | 151.3 | 40.5 | 33.6 | 39.4 | 89.7 | 130.9 |
| Second | 57.5 | 55.4 | 86.1 | 165.3 | 88.5 | 47.9 | 46.2 | 71.7 | 137.7 | 73.7 |
| Third | 62.5 | 66.9 | 95.4 | 158.2 | 43.3 | 49.4 | 52.8 | 75.4 | 125.0 | 34.2 |
| Fourth | 40.0 | 49.8 | 57.4 | 105.9 | 28.9 | 39.0 | 48.5 | 55.9 | 103.2 | 28.2 |
| Fifth (top 20%) | 18.2 | 26.3 | 25.8 | 51.3 | 7.9 | 31.0 | 44.7 | 44.0 | 87.2 | 13.4 |
| Total | 225.0 | 237.3 | 310.2 | 584.4 | 319.8 | 207.8 | 225.9 | 286.3 | 542.8 | 280.4 |
| First:fifth ratio | 2.6 | 1.5 | 1.8 | 2.0 | 19.3 | 1.3 | 0.8 | 0.9 | 1.0 | 9.8 |
| **Total life years gained (in thousands)** | | | | | | | | | | |
| First (bottom 20%) | 392.0 | 321.0 | 375.9 | 873.2 | 1261.6 | 339.2 | 277.8 | 325.3 | 755.6 | 1091.7 |
| Second | 448.8 | 464.7 | 743.4 | 1464.4 | 773.2 | 373.8 | 387.0 | 619.2 | 1219.7 | 644.0 |
| Third | 504.0 | 573.6 | 822.4 | 1334.2 | 366.6 | 398.1 | 453.0 | 649.6 | 1053.8 | 289.6 |
| Fourth | 326.8 | 421.8 | 487.2 | 885.2 | 244.3 | 318.5 | 411.1 | 474.9 | 862.8 | 238.1 |
| Fifth (top 20%) | 147.0 | 216.6 | 216.0 | 416.8 | 65.9 | 250.0 | 368.4 | 367.3 | 708.9 | 112.1 |
| Total | 1818.6 | 1997.6 | 2645.0 | 4973.7 | 2711.6 | 1679.6 | 1897.3 | 2436.3 | 4600.8 | 2375.5 |
| First:fifth ratio | 2.7 | 1.5 | 1.7 | 2.1 | 19.1 | 1.4 | 0.8 | 0.9 | 1.1 | 9.7 |
| **Total deaths averted (in thousands)** | | | | | | | | | | |
| First (bottom 20%) | 19.6 | 16.2 | 18.7 | 43.9 | 62.9 | 16.9 | 14.0 | 16.1 | 38.0 | 54.5 |
| Second | 22.9 | 23.2 | 36.8 | 71.8 | 38.1 | 19.0 | 19.4 | 30.6 | 59.8 | 31.7 |
| Third | 25.4 | 28.4 | 40.8 | 66.5 | 18.3 | 20.1 | 22.5 | 32.3 | 52.6 | 14.5 |
| Fourth | 16.3 | 21.1 | 24.3 | 44.4 | 12.1 | 15.9 | 20.6 | 23.7 | 43.3 | 11.8 |
| Fifth (top 20%) | 7.4 | 10.9 | 11.0 | 20.8 | 3.3 | 12.6 | 18.5 | 18.7 | 35.4 | 5.6 |
| Total | 91.5 | 99.8 | 131.5 | 247.5 | 134.7 | 84.5 | 94.9 | 121.3 | 229.0 | 118.0 |
| First:fifth ratio | 2.6 | 1.5 | 1.7 | 2.1 | 19.2 | 1.3 | 0.8 | 0.9 | 1.1 | 9.8 |
| **Treatment cost averted (MX$ (PPP$), in billions)** | | | | | | | | | | |
| First (bottom 20%) | 1.4 (0.2) | 1.1 (0.1) | 1.3 (0.1) | 2.8 (0.3) | 3.9 (0.4) | 1.2 (0.1) | 0.9 (0.1) | 1.1 (0.1) | 2.4 (0.3) | 3.4 (0.4) |
| Second | 1.6 (0.2) | 1.7 (0.2) | 2.7 (0.3) | 4.8 (0.5) | 2.7 (0.3) | 1.3 (0.1) | 1.4 (0.2) | 2.2 (0.2) | 4.0 (0.4) | 2.2 (0.2) |
| Third | 1.9 (0.2) | 2.1 (0.2) | 3.2 (0.3) | 4.9 (0.5) | 1.3 (0.1) | 1.5 (0.2) | 1.7 (0.2) | 2.5 (0.3) | 3.9 (0.4) | 1.0 (0.1) |
| Fourth | 1.1 (0.1) | 1.6 (0.2) | 1.9 (0.2) | 3.3 (0.4) | 0.9 (0.1) | 1.1 (0.1) | 1.6 (0.2) | 1.9 (0.2) | 3.2 (0.3) | 0.8 (0.1) |
| Fifth (top 20%) | 0.5 (0.1) | 0.8 (0.1) | 0.9 (0.1) | 1.4 (0.1) | 0.2 (0.03) | 0.9 (0.1) | 1.3 (0.1) | 1.5 (0.2) | 2.4 (0.3) | 0.4 (0.04) |
| Total | 6.6 (0.7) | 7.3 (0.8) | 10.0 (1.1) | 17.2 (1.9) | 9.0 (1.0) | 6.1 (0.7) | 6.9 (0.7) | 9.3 (1.0) | 15.9 (1.7) | 7.9 (0.9) |
| First:fifth ratio | 2.7 | 1.4 | 1.4 | 2.0 | 16.4 | 1.4 | 0.7 | 0.7 | 1.0 | 8.4 |
| **Additional tax revenue per year (MX$ (PPP$), in billions)** | | | | | | | | | | |
| First (bottom 20%) | 0.07 (0.01) | 0.05 (0.01) | 0.13 (0.01) | 0.10 (0.01) | 0.11 (0.01) | 0.14 (0.02) | 0.11 (0.01) | 0.20 (0.02) | 0.17 (0.02) | 0.20 (0.02) |
| Second | 0.33 (0.04) | 0.15 (0.02) | 0.32 (0.03) | 0.28 (0.03) | 0.08 (0.01) | 0.47 (0.05) | 0.23 (0.02) | 0.50 (0.05) | 0.45 (0.05) | 0.13 (0.01) |
| Third | 0.48 (0.05) | 0.34 (0.04) | 0.53 (0.06) | 0.67 (0.07) | 0.15 (0.02) | 0.65 (0.07) | 0.47 (0.05) | 0.77 (0.08) | 0.93 (0.10) | 0.20 (0.02) |
| Fourth | 0.72 (0.08) | 0.70 (0.08) | 1.16 (0.13) | 1.29 (0.14) | 0.30 (0.03) | 0.73 (0.08) | 0.71 (0.08) | 1.18 (0.13) | 1.31 (0.14) | 0.30 (0.03) |
| Fifth (top 20%) | 1.07 (0.12) | 1.27 (0.14) | 1.76 (0.19) | 2.22 (0.24) | 0.28 (0.03) | 0.93 (0.10) | 1.10 (0.12) | 1.52 (0.16) | 1.93 (0.21) | 0.24 (0.03) |
| Total | 2.68 (0.29) | 2.52 (0.27) | 3.91 (0.42) | 4.56 (0.49) | 0.91 (0.10) | 2.92 (0.31) | 2.63 (0.28) | 4.18 (0.45) | 4.79 (0.52) | 1.07 (0.12) |
| First:fifth ratio | 0.07 | 0.04 | 0.07 | 0.05 | 0.40 | 0.15 | 0.10 | 0.13 | 0.09 | 0.82 |
| **Number of people avoiding poverty (in thousands)** | | | | | | | | | | |
| First (bottom 20%) | 3.5 | 2.6 | 2.9 | 6.3 | 5.8 | 3.0 | 2.2 | 2.5 | 5.4 | 5.0 |
| Second | 20.2 | 22.1 | 32.6 | 62.1 | 35.9 | 16.8 | 18.4 | 27.1 | 51.8 | 29.9 |
| Third | 9.2 | 8.4 | 23.1 | 49.7 | 14.0 | 7.3 | 6.6 | 18.2 | 39.3 | 11.1 |
| Fourth | 2.5 | 3.0 | 3.5 | 5.9 | 1.9 | 2.4 | 3.0 | 3.4 | 5.8 | 1.9 |
| Fifth (top 20%) | 0.0 | 0.0 | 0.4 | 0.8 | 0.2 | 0.0 | 0.0 | 0.6 | 1.3 | 0.3 |
| Total | 35.3 | 36.1 | 62.4 | 124.8 | 57.9 | 29.5 | 30.2 | 51.9 | 103.6 | 48.2 |
| First:fifth ratio | — | — | 7.6 | 8.0 | 28.3 | — | — | 3.9 | 4.1 | 14.4 |
| **Number of people avoiding catastrophic health expenditures (in thousands)** | | | | | | | | | | |
| First (bottom 20%) | 18.2 | 14.1 | 15.9 | 35.8 | 52.3 | 15.7 | 12.2 | 13.8 | 31.0 | 45.2 |
| Second | 20.2 | 22.2 | 32.6 | 62.1 | 35.9 | 16.9 | 18.5 | 27.1 | 51.8 | 29.9 |
| Third | 24.4 | 27.3 | 39.2 | 63.9 | 17.6 | 19.3 | 21.6 | 31.0 | 50.5 | 13.9 |
| Fourth | 14.7 | 20.8 | 23.6 | 42.8 | 11.6 | 14.3 | 20.3 | 23.0 | 41.7 | 11.3 |
| Fifth (top 20%) | 5.8 | 8.6 | 9.9 | 16.6 | 3.2 | 9.8 | 14.6 | 16.9 | 28.2 | 5.4 |
| Total | 83.3 | 93.0 | 121.3 | 221.2 | 120.6 | 76.0 | 87.2 | 111.8 | 203.1 | 105.8 |
| First:fifth ratio | 3.1 | 1.6 | 1.6 | 2.2 | 16.4 | 1.6 | 0.8 | 0.8 | 1.1 | 8.4 |

Notes: Scenario 1 uses the same elasticity as the main scenario for quintiles 1 and 5 (-0.635 and -0.122, respectively), but considers the estimate from Supplementary Table A3 for quintile 3 (-0.479). Scenario 2 uses the same elasticity as the main scenario for quintile 3 (-0.379) but considers a lower elasticity (in absolute terms) for quintiles 1 and 5 (-0.550 and -0.208, respectively). The modelled scenario considers a one-peso tobacco tax increase, roughly equivalent to a 44% increase in price. The benefits of the tax increase would be observed during the lifetime of the current smoking cohort, except for the additional tax revenue that would be annual. PPP$ = International dollars adjusted for purchasing power parity 2019. MX$ = Mexican pesos of 2020.

**Figure A1. Sensitivity analysis for health and financial outcomes by varying price elasticities**

**Life years gained (in thousands)**


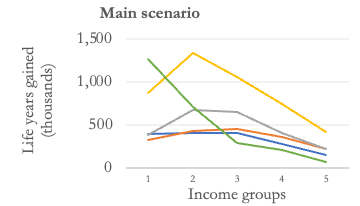

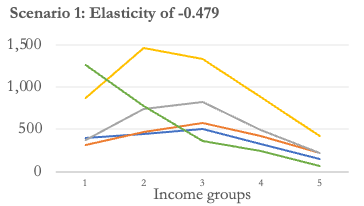

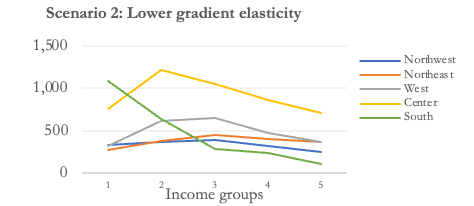

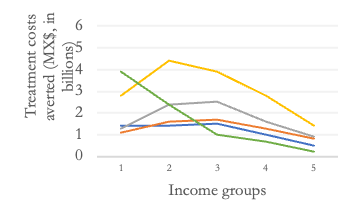

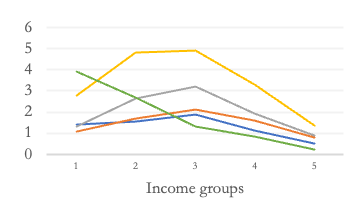

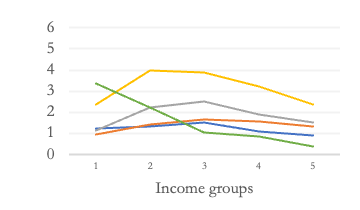

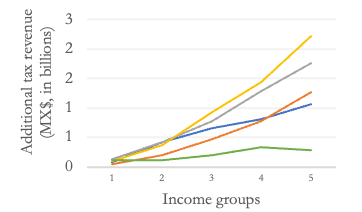

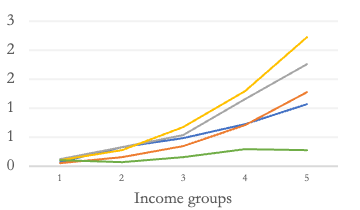

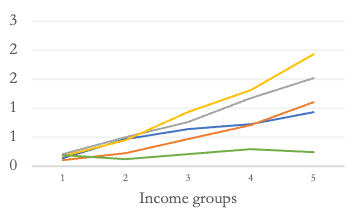


**Treatment costs averted (MX$, in billions)**

**Additional tax revenue per year (MX$, in billions)**

Notes: These graphs are based on Supplementary Table A6 above. Scenario 1 uses the same elasticity as the main scenario for quintiles 1 and 5 (-0.635 and -0.122, respectively), but considers the estimate from Supplementary Table A3 for quintile 3 (-0.479). Scenario 2 uses the same elasticity as the main scenario for quintile 3 (-0.379 but considers a lower elasticity (in absolute terms) for quintiles 1 and 5 (-0.550 and -0.208, respectively). The modelled scenario considers a one-peso tobacco tax increase. MX$ = Mexican pesos of 2020.

**Table A7. Checking the internal consistency of the model: national estimates versus sum of regional estimates**

| **Outcomes by income group** |  | **National estimates**  **(A)** |  | **Sum of regional estimates**  **(B)** | **(A) – (B)** |  |
| --- | --- | --- | --- | --- | --- | --- |
| **Number of smokes who quit smoking (in thousands)** | | | | | |  |
| First (bottom 20%) |  | 386.0 |  | 386.0 | 0.0 |  |
| Second |  | 411.9 |  | 411.9 | 0.0 |  |
| Third |  | 336.7 |  | 336.7 | 0.0 |  |
| Fourth |  | 234.8 |  | 234.8 | 0.0 |  |
| Fifth (top 20%) |  | 129.5 |  | 129.5 | 0.0 |  |
| Total |  | 1,499.0 |  | 1,499.0 | 0.0 |  |
| First:fifth ratio |  | 3.0 |  | 3.0 | 0.0 |  |
| **Total life years gained (in thousands)** | | | | | |  |
| First (bottom 20%) |  | 3,223.6 |  | 3,223.6 | 0.0 |  |
| Second |  | 3,542.6 |  | 3,542.6 | 0.0 |  |
| Third |  | 2,844.1 |  | 2,844.1 | 0.0 |  |
| Fourth |  | 1,969.1 |  | 1,969.1 | 0.0 |  |
| Fifth (top 20%) |  | 1,062.2 |  | 1,062.2 | 0.0 |  |
| Total |  | 12,641.7 |  | 12,641.7 | 0.0 |  |
| First:fifth ratio |  | 3.0 |  | 3.0 | 0.0 |  |
| **Total deaths averted (in thousands)** | | | | | |  |
| First (bottom 20%) |  | 161.2 |  | 161.2 | 0.0 |  |
| Second |  | 175.4 |  | 175.4 | 0.0 |  |
| Third |  | 141.8 |  | 141.8 | 0.0 |  |
| Fourth |  | 98.4 |  | 98.4 | 0.0 |  |
| Fifth (top 20%) |  | 53.3 |  | 53.3 | 0.0 |  |
| Total |  | 630.1 |  | 630.1 | 0.0 |  |
| First:fifth ratio |  | 3.0 |  | 3.0 | 0.0 |  |
| **Treatment cost averted (MX$ (PPP$), in billions)** | | | | | |  |
| First (bottom 20%) |  | 10.4 (1.1) |  | 10.5 (1.1) | 0.1 |  |
| Second |  | 12.3 (1.3) |  | 12.2 (1.3) | 0.0 |  |
| Third |  | 10.6 (1.1) |  | 10.6 (1.1) | 0.1 |  |
| Fourth |  | 7.4 (0.8) |  | 7.4 (0.8) | 0.0 |  |
| Fifth (top 20%) |  | 3.8 (0.4) |  | 3.8 (0.4) | 0.0 |  |
| Total |  | 44.4 (4.6) |  | 44.6 (4.6) | 0.1 |  |
| First:fifth ratio |  | 2.7 |  | 2.7 | 0.0 |  |
| **Additional tax revenues per year (MX$ (PPP$), in billions)** | | | | | |  |
| First (bottom 20%) |  | 0.47 (0.05) |  | 0.46 (0.05) | 0.01 |  |
| Second |  | 1.43 (0.15) |  | 1.50 (0.16) | 0.07 |  |
| Third |  | 3.05 (0.33) |  | 3.02 (0.33) | 0.03 |  |
| Fourth |  | 4.57 (0.49) |  | 4.63 (0.50) | 0.06 |  |
| Fifth (top 20%) |  | 6.70 (0.72) |  | 6.59 (0.71) | 0.11 |  |
| Total |  | 16.22 (1.75) |  | 16.21 (1.75) | 0.01 |  |
| First:fifth ratio |  | 0.07 |  | 0.07 | 0.00 |  |
| **Number of people averting poverty (in thousands)** | | | | | |  |
| First (bottom 20%) |  | 20.6 |  | 21.0 | -0.4 |  |
| Second |  | 157.8 |  | 157.3 | 0.5 |  |
| Third |  | 87.0 |  | 82.4 | 4.5 |  |
| Fourth |  | 14.4 |  | 14.0 | 0.4 |  |
| Fifth (top 20%) |  | 2.0 |  | 1.4 | 0.6 |  |
| Total |  | 281.7 |  | 276.1 | 5.6 |  |
| First:fifth ratio |  | 10.3 |  | 15.0 | -4.7 |  |
| **Number of people avoiding catastrophic health expenditures (in thousands)** | | | | | | |
| First (bottom 20%) |  | 133.7 |  | 136.3 | -2.6 |  |
| Second |  | 157.8 |  | 157.4 | 0.3 |  |
| Third |  | 136.1 |  | 136.1 | 0.0 |  |
| Fourth |  | 94.5 |  | 94.5 | 0.1 |  |
| Fifth (top 20%) |  | 45.0 |  | 44.1 | 0.9 |  |
| Total |  | 567.1 |  | 568.4 | -1.3 |  |
| First:fifth ratio |  | 3.0 |  | 3.1 | -0.1 |  |

Notes: The modelled scenario considers a one-peso tobacco tax increase, roughly equivalent to 44% increase in price. The benefits of the tax increase would be observed during the lifetime of the current smoking cohort, except for the additional tax revenue that would be annual. PPP$ = International dollars adjusted for purchasing power parity 2019. MX$ = Mexican pesos of 2020. National estimates (A) are obtained by running the model with national inputs (see Supplementary Table A3). The sum of regional estimates (B) corresponds to the last column (Total) of Table 2.

**Figure A2. Share of the benefits of a one-peso tax increase that would correspond to the bottom and top quintiles**

Notes: Estimates are based on Table 2.
